# Some New Sedatives: Their Value Compared with That of Older Drugs

**Published:** 1907-09

**Authors:** Arthur Foxwell

**Affiliations:** Professor of Thereapeutics in the University of Birmingham, and Senior Physician to the Queen’s Hospital, Birmingham


### SELECTED ARTICLES.

### Some New Sedatives: Their Value Compared with That of Older Drugs.

By ARTHUR FOXWELL, M.A., M.D. (Cantab.), F.R.C.P.
Professor of Thereapeutics in the University of Birmingham, and Senior Physician to the Queen's Hospital, Birmingham.

[*Folia Therapeutica, July, 1907.*]

IN this paper I make an attempt to compare the clinical values of some of our more recent sedatives. I use the word sedative rather than hypnotic because often the more valuable use of these drugs is to procure a sedation of undue nervous irritability during the waking hours of the day rather than the induction of sleep. Sedative is the larger term, and includes the smaller, hypnotic.

Naturally, I can only speak with advantage of those drugs of which I have direct personal experience: this greatly limits my field of view, but, the better to indicate the position of these newer drugs in the general scale of sedatives, I shall include one or two older ones of whose value I, at any rate, have no doubt.

The drugs whose virtues I am about to compare are:—Codein, dionin, heroin, morphine: chloral, butyl-chloral, amylene-chloral: the bromides, bromural, bromocarpine, bromidia: paraldehyde: sulphonal, trional, veronal: strychnine, mercury.

If one considers these generally, without any reference to the special conditions of the patient, merely in accordance with their power of inducing sleep in a quite healthy person—then I would place them in this order, beginning with the feeblest:—

1. Strychnine and mercury: no action.
2. Codein, butyl-chloral, bromocarpine.
3. Amylene-chloral, bromural, sulphonal, bromide of ammonium, bromide of sodium.
4. Dionin, bromide of potassium, bromidia, paraldehyde; trional.

5. Heroin, chloral hydrate, veronal.
6. Morphine; the dose given being the average dose for each drug.

But one example is sufficient to show the unscientific nature of this classification and the great unwisdom of exhibiting any sedative or soporific without having previously made a thorough diagnosis of your patient. Mr. ——, aged 56, suffering from syphiltic phthisis of long duration, had had persistent insomnia for nine months, since the death of his wife. His brain was incoherently active in the night, but drowsy during the day: at night he rarely got more than two hours' sleep. He had tried many soporifics, including opium, but all in vain. His heart had both its ventricles dilated, his arteries were much thickened, and his tension very high. It was evident that his heart was failing, being unable to cope with his thick vessels and the high tension, the insomnia of cerebral anemia resulting. He was already on a cardiac tonic, so I merely gave him a quarter of a grain of calomel to be taken thrice daily. In a week he came again: his tension was very slightly above normal, and he had slept every night from eleven to four, on two nights dosing off again after four.

I will now briefly indicate the special value of the members of each group.

Group I.—To the exhausted anemic, or overworked debilitated person, strychnine is the hypnotic *par excellence*: a full dose, *m* v. to *m* vij., given at bedtime, bringing sound and long lasting sleep followed by a reinvigorated awakening. The feeble, too easily irritated nerve cells, are toned up to a healthy disregard of the petty impulses—subjective as well as objective—which previously kept them on the rack. In medicinal doses, strychnine is far more a tonic than a stimulant, that is, it raises the nerve cell to a *permanent* level of greater efficiency.

Mercury stands in the same relation to undue high tension that strychnine does to nervous debility. But it rarely acts so quickly: twenty-four hours usually being needed to produce its effect. It has to remove from the blood the poison which is producing the unduly high tension. I say *unduly* high, for if the tension be only such as is necessitated by thickened arteries, and not one due to any toxin, then mercury will have no effect upon it, and sedation will not result.

Thus both strychnine and mercury do not act as simple sedatives by covering, so to speak, with a cloak of cotton wool, a too irritable nervous system, but they remove the cause of the irritability, and allow the system to act normally.

Group II.—Codein as a general sedative has little or no power. No doubt it acts as a soporific in some cases of irritable cough with insomnia, but this action is probably due merely to its soothing action on the cough.

Butyl-chloral is another local sedative: it gives most excellent results in neuralgia of the fifth nerve, and in toothache, especially if this be neuralgic in character. For this purpose I have often given it in five grain doses, every half-hour till relief arrived, or twenty-five grains had been taken, but though it has frequently brought relief, I have failed to note any general action as a soporific, the patient's brain being just as active as before its exhibition.

Dr. Armstrong, of Buxton, first drew my attention to bromocarpin [KBr. 10 per cent., pilocarpine hydrobromide ½ per cent.]: I have used it frequently since, where it was important that the sedative should not stop the secretions but rather increase them—*e.g.*, in gouty people. I think it achieves its purpose. I have given it in drachm doses thrice daily. As my patients were nearly all well enough to go about their daily duties it will require a far more extended observation to make any dogmatic statement concerning it. Dr. Armstrong, who had used it a great deal, spoke very highly of it.

Group III.—Amylene-chloral.—My experience with this drug has been almost limited to its sedative action: but on the one or two occasions on which I have given it as a hypnotic it has done well. As a sedative I give *m* 15 of the 50 per cent. solution *t.d.*; as an hypnotic *m* 30 to *m* 50. In one or two neurotic women it has acted well as a sedative, when bromides, trional, and even chloral hydrate have failed. I do not think it induces a habit: at any rate one lady who took it for two or three months gave it up gradually of her own accord without any effort. I think highly of it.

Bromural [monobrom—isovalerianyl—urea] has been tried a good deal in Germany. Bromine is not dissociated from it in the body, and its sedative effect is supposed to be due to the isopropyl group being linked with the valerianic acid: at the same time one must remember that similar compounds containing iodine or chlorine instead of bromine have no sedative action. It is claimed to have no ill-effect; that the sleep obtained by it is natural; that the patient wakes up feeling as fresh and clear as after natural sleep. I have used it a good deal during the last three months, and my own experience bears out the validity of these claims. Its action is a short lived one—two to five hours—it is, therefore, not a good drug to keep up a continuous sedative effect with, and I have only tried it for inducing sleep at night. In all mild cases I have found it uniformly successful: sleep came within the hour and lasted till the usual time of awaking. In two instances where there was a continuing source of irritation the patients woke in four hours feeling heavy and headachy; but where the sleep lasted right through the night, no such effect was noted. This bears out the contention of the short duration of its action, the natural sleep succeeding this action allowing time for any drowsiness, &c., due to the drug, to wear off. For simple wakefulness, then, it is peculiarly suitable and I know no better drug. It also combats successfully mild forms of irritation, and is said to have even induced sleep in sciatica and tabes with lightning pains. But in severe delirium of fever, uremic conditions, the restlessness of failing heart, great mental excitement, &c., it fails. Its action is prompter than that of the bromides, sleep usually coming within the hour. The dose I have used is one or two tablets, each containing 0.3 grm.; oftener two tablets are needed. I think bromural has come to stay.

Sulphonal is an uncertain drug, and for this reason I have not used it much of late years. Sometimes it acts perfectly and leaves no trace behind: at others it produces no real sleep, but a persistent drowsiness, which may last 24 hours or more, temporarily spoiling the appetite, and making active mental work an impossibility. My usual dose is 25 grains, from which I have seen no serious ill effects.

The bromides are our most certain drugs for maintaining continuous sedation. Their action is slower in onset than the other drugs here considered: one to two hours being necesessary to obtain their full effect. But in my experience they are without a rival in their power of maintaining mental quiet for weeks or months with little or no deleterious result, and with no tendency to induce a habit. For this purpose, five grains thrice daily is the dose I usually employ. The dose necessary to produce sleep—20 to 60 grs.—is so large, that its action is not seldom prolonged into the succeeding day, and renders the patient less apt for his work.

Group IV.—Bromide of potassium I have placed here rather than in Group III. as its action is distinctly stronger than that of the other two bromides, though of the same nature. This extra activity enables it to be used as a hypnotic, though the liability to be drowsy during the next day is still a drawback. Bromidia [bromide of potassium, gr. 10: chloral hydrate, gr. 10: succus hyoscyami *m* 10: tr. cann. indicæ *m* 2½ to the drachm] is a very reliable hypnotic of considerable strength: a drachm being a sufficient dose in most ordinary cases of insomnia. It acts more powerfully than the mere mixture of the chloral and bromide: at the same time it has less after-effect, and much less tendency to induce a habit than chloral alone. In doses of *m* 10 to *m* 20

*t.d.* it is very useful for continuous sedation, where small doses of a bromide alone are insufficient.

Dionine—ethyl-morphine hydrochloride—was introduced to English practice in 1899, about the same date as heroin. But, so far, it has not achieved anything like the popularity of the latter drug. It is much weaker in its action—gr. 1-20 heroin—gr. 1-5 dionine. But its exhibition is rarely followed by sickness or other malaise—I found this sequela three times in sixty trials. It does not lessen the respiratory activity: according to the experiments of Winternitz and Damisch it increases the respiratory exchange by 1 to 1½ litres per minute; whereas they found heroin lessened this exchange like morphine. In a case of acute bronchitis I gave gr. ¼ dionine: this was followed by deep sleep throughout the night, with drowsiness the next morning, but there was no evidence of any bad effect on the respiration. In many cases the relief given to cough has not been marked, even where several hours of sleep were obtained: other observers find it as great a reliever of cough as heroin, especially of whooping cough and the irritable cough of phthisis. It is somewhat difficult to fit in this sedative action on cough with its action as a respiratory stimulant and further observations are needed on this latter action at any rate. It is still more difficult to agree with the dictum of Bancke, " as dionine reduces the irritability of the respiratory tract, without impairing its function, and promotes expectoration, it may be used in all diseases of the respiratory organs accompanied with irritation," for it is generally admitted that dionine produces its effect by its action on the centres in the medulla.

Dionine has not the immediate exhilarating effect which is so dangerous a quality of morphine: it does not tend to induce a habit, and has been successfully used to combat the abuse of morphine.

To sum up—the hypnotic and analgesic effect is a great deal stronger than that of codeine, but far inferior to that of morphine or heroin, in the doses at present used. Like codeine, it seems to be almost free from bad after-effects, and capable of being used freely and safely in respiratory and renal disease. Its usual dose is gr. ¼ to gr. ½, but the smaller dose frequently only brings about 2 or 3 hours' sleep, more than 4 being the exception (in only one-third of my cases).

I would add that it is a white crystalline powder, freely soluble in water, unirritating when given subcutaneously, and therefore easy of administration.

Paraldehyde is a thoroughly reliable hypnotic of moderate strength, with no bad after-effects. When it first came out I tried it a great deal in cases of restless insomnia due to severe aortic valve disease: it did not always succeed in obtaining sleep, but it never depressed the circulation, nor produced other malaise, so far as I could discover. That it should not always succeed in such cases is not to be wondered at, for aortic insomnia is a difficult one to remedy with anything short of morphine. The great objection to paraldehyde is its nauseous flavor, which persists for 24 hours, whether given by mouth or rectum. The dose is ʒj, and a larger one is but little more effective.

Trional. Of the three drugs, sulphonal, trional, and tetranol, trional has best maintained its reputation. I have known it, in a case of hemorrhagic restlessness, act well, repeatedly, in 10 grain doses, after morphine and others had failed: we have all known it fail; and sometimes it produces alarming symptoms. But on the whole it is a reliable hypnotic of moderate strength and, in doses of 10 to 15 grains, rarely produces any untoward symptoms.

Group V.—Heroin.—Heroin and dionine were put on the English market about the same time—1898-1899. Dionine, for some reason or other, missed fire at first, but heroin caught on at once and has never looked back since. It certainly lessens respiratory exchange, though much less so than an equivalent dose of morphine. It soothes the respiratory tract more than the other systems of the body.. It is to some extent an analgesic, though far less so than its morphine equivalant. It is not free from the opiate malaise, though this is comparatively slight; and its reduction of the renal secretions has to be carefully borne in mind.

Heroin gr. 1-20—morphine gr. 1-10—dionine gr. 1-5, is about the right ratio of dosage to produce the same general hypnotic effect; but heroin would have the best effect upon cough, and morphine would prove the best general analgesic, whilst dionine would least upset the body functions.

Chloral hydrate has gone, I think, too much out of fashion at present, just as it was too much in fashion 25 years ago. It is more to be commended as a sedative in gr. 5 doses *t.d.,* than as a hypnotic in doses of gr. 20 and upwards. Given thus, as one ingredient in a tonic prescription, it runs no danger of inducing a habit and acts excellently. In larger doses, as a hypnotic, it is apt to depress the heart, but I think it is only when given continuously that it causes anxiety on this score, or if the patient's heart be already greatly weakened by alcohol or fatty degeneration. When given for long periods no doubt it greatly lowers the nervous system and thus induces cardiac failure.

Veronal, taken altogether, is the most satisfactory hypnotic I know. Its action is sure, powerful, and unaccompanied by untoward complications. I gave a chronic bronchitic with a feeble dilated heart, a woman of 60, gr. 5 *t.d.*, for restlessness, troublesome cough and asthmatic breathing: by an oversight it was continued for five days: the patient became semi-comatose, but the respiration was quieter and fuller and the pulse stronger: there was no stertor, the condition being more nearly allied to trance, and from it she could always be momentarily roused. Cerebral thrombosis was feared, but on removing the drug the normal waking state slowly returned and, with it, her condition was in every way greatly improved. As much as 65 grains was taken by a melancholic woman—a semi-comatose state, with stertor lasting 24 hours, resulted, and was not entirely recovered from for six days, but the pulse remained good throughout. Rarely giddiness, headache and vomiting have been noticed, as well as transient eruptions, but no serious complications.

As a simple hypnotic, 3 grains is a fair dose to start with: very seldom, except in mental cases, will more than 7 or 8 be required. But writers differ very widely as to the dose; Lilienfeld [Berliner Klin. Wochenschr. No. 20, 1903], says that only in doses of 15 grains and over is drowsiness felt the next day, whereas in two or three instances I have known rather annoying drowsiness to result from one-third this dose. Drs. Berent [Therap. Monatshefte, June, 1903], and Würth [Psychiatr-Neurolg. Wochenschr. No. 9, June, 1903], both think it equal to double the quantity of trional. This seems to me a very fair judgment of its strength; and it has the great advantage that you can give as large a dose of it as of trional, or even a larger one. Prof. Bartholow recommends trying the two together—two parts veronal to one part trional—he says they both have a cumulative action, so that if you begin with a full dose you can lessen the amount in succeeding ones. Others deny this cumulative action. Cumulative action is always a difficult thing to determine: my own experience is as yet too limited to make a decided statement, but I am inclined to agree with Prof. Bartholow; though I do not think there is any danger of a *sudden denouement* of its accumulation, such as one associates with digitalis, but only an increasing effect from continuing the same dose. Veronal should give excellent results as a sedative, and in the few cases where I have tried it in doses of 1½ to 2 grs., *t.-d.*, I have not been disappointed. But one must try it for several months on the same patient, and on several patients, before any dogmatic statement can be made.

It is far more soluble in hot liquids than cold: hence when a speedy action is required it is best given in hot tea, milk, or water. Given thus, it should induce sleep in half an hour. The sleep is quiet and natural and, if the dose be small, no unpleasant heaviness is experienced on awaking. At the same time, it must be admitted, it is much more likely to cause this sequela than such a drug as bromural: in fact, so far as after unpleasantness is concerned, bromural is easily first of all drugs of which I have experience.

Morphine, as an analgesic, stands alone: the others, if they produce analgesia, do so only as a part of a general drowsiness and dulling of sensation: morphine alone can assuage pain, and yet leave the intellect clear and alert. But for insomnia, apart from pain, not only are its after-effects often so unpleasant that the patient prefers sleeplessness, but its hypnotic power is not very constant, and even half a grain may produce a pleasant wakefulness instead of sleep.

